# Reply: sex-related variations in platelet reactivity

**DOI:** 10.1093/ehjcvp/pvaf054

**Published:** 2025-07-21

**Authors:** Mattia Galli, Dominick J Angiolillo, Fabio M Pulcinelli

**Affiliations:** Maria Cecilia Hospital, GVM Care & Research, Cotignola 48033, Italy; Department of Medical-Surgical Sciences and Biotechnologies, Sapienza University of Rome, Latina 04100, Italy; Division of Cardiology, University of Florida College of Medicine, Jacksonville, FL, USA; Department of Experimental Medicine, Sapienza University of Rome, Viale Regina Elena 324, Rome 00161, Italy

We thank Prof. Marco Cattaneo for the thoughtful letter regarding our recent study, the largest pharmacodynamic investigation to date examining sex-related differences in platelet reactivity among patients with and without antiplatelet therapy.^1^ The letter underscores the relevance of the topic of our investigation aimed at elucidating the biological underpinnings of sex-specific responses to antiplatelet agents, an issue of growing importance in contemporary cardiovascular medicine.^2^

Prof. Cattaneo raises the possibility that hematocrit-related artefacts may have influenced the platelet aggregation results.^3^ Specifically, it is suggested that lower haematocrit levels in women than in men could alter citrate distribution, leading to higher plasma calcium concentrations and potentially contributing to the observed sex differences in platelet reactivity. It is worth noting that the mentioned haematocrit-based adjustment is not part of standard operating procedures and accordingly not routinely applied in the vast majority of contemporary studies.^4–9^ This hypothesis is primarily supported by a dated study from 1980 involving 11 male and 11 female healthy volunteers with a mean age of 32 years.^10^ While mechanistically interesting, the study presents several limitations: the sample size is small, the population is significantly younger and healthier than typical cardiovascular cohorts, and the methodological details—including the type and concentration of platelet agonists used—are not full defined and most likely not comparable to current day protocols, including ours. The reproducibility of light transmission aggregometry is highly dependent on a number of parameters, including the choice and concentration of agonist, operator expertise, and instrument calibration.^4^ For instance, the 1980 study used a platelet count adjustment technique (reconstitution of platelet-rich plasma to 200 000/μL using autologous platelet-poor plasma) not commonly employed in more recent studies—including in our analysis.^5–9,11^ Moreover, in our analysis, females under 50 years of age exhibited lower platelet reactivity compared with those over 50, providing an additional explanation for the discrepancy with the 1980 study, which included a younger population of healthy volunteers.^1^ Collectively, given the substantial differences in methodology, population (mean age = 32 years vs. 64 years), and sample size (*n* = 22 vs. *n* = 5687), the two studies cannot be compared. Our large-scale dataset likely offers sufficient statistical power to detect even more modest biological differences, which are typically more pronounced in the post-menopausal female population.^12^

Another important point is that the studies cited by Prof. Cattaneo are purely laboratory-based without clinical correlates. No *in vitro* study alone can offer conclusive evidence in this regard, and laboratory-based speculations must be interpreted cautiously. From a clinical standpoint, the primary concern lies in determining whether the observed sex differences in platelet reactivity assessed ex vivo translate into meaningful adverse outcomes. Laboratory adjustments may potentially eliminate real biological differences between sexes and may inadvertently conceal factors that help explain observed variations in treatment response and their impact on clinical outcomes. These laboratory adjustments, in fact, can interfere with measured levels of platelet reactivity and thus should be avoided, as also recommended by Cattaneo *et al*.^11^ To date, numerous ex vivo studies have shown that levels of on-treatment platelet reactivity—measured using various assays—is associated with adverse clinical outcomes, both ischaemic and haemorrhagic, particularly in patients undergoing percutaneous coronary intervention (PCI).^13^ Moreover, antiplatelet therapy guided by the use of platelet function testing has been shown to improve outcomes in patient undergoing PCI.^14^ Accordingly, while not recommended for routine use, platelet function testing does have a role in specific clinical scenarios, as supported by international guidelines and consensus documents.^4,15^ Thus, we respectfully disagree with the statement made in his letter alluding to the lack of clinical benefit associated with monitoring antiplatelet therapy with platelet function tests.

Finally, it should be noted that our results, in larger scale, are consistent with a growing body of meticulously conducted studies that report similar sex-related differences in platelet reactivity.^7,8^ Moreover, our findings are aligned with recent randomized controlled trials suggesting that women may experience different clinical effects from antiplatelet therapies compared with men.^16,17^ While our study results are not definitive and require further research, it does offer a potential mechanistic explanation for the differences in sex-related outcomes in patients treated with antiplatelet therapy.

## Data Availability

Data sharing is not applicable to this article as no new data were created or analysed in this manuscript.

## References

[1] Galli M, Terracina S, Schiera E, De Corci S, Sangiorgi D, Mancone M, Frati L, Sciarretta S, Angiolillo DJ, Pulcinelli FM. Sex-related variations in platelet reactivity in presence or absence of antiplatelet therapy. Eur Heart J Cardiovasc Pharmacother 2025;11:509–517. 40366913 10.1093/ehjcvp/pvaf034 PMC12450592

[2] Laborante R, Borovac JA, Galli M, Rodolico D, Ciliberti G, Restivo A, Cappannoli L, Arcudi A, Vergallo R, Zito A, Princi G, Leone AM, Aurigemma C, Romagnoli E, Montone RA, Burzotta F, Trani C, D’Amario D. Gender-differences in antithrombotic therapy across the spectrum of ischemic heart disease: time to tackle the Yentl syndrome? Front Cardiovasc Med 2022;9:1009475. 36386309 10.3389/fcvm.2022.1009475 PMC9659635

[3] Cattaneo M. Sex-related differences in platelet aggregation. Eur Heart J Cardiovasc Pharmacother 2025;11:518–519. 40671586 10.1093/ehjcvp/pvaf052 PMC12450590

[4] Angiolillo DJ, Galli M, Alexopoulos D, Aradi D, Bhatt DL, Bonello L, Capodanno D, Cavallari LH, Collet JP, Cuisset T, Ferreiro JL, Franchi F, Geisler T, Gibson CM, Gorog DA, Gurbel PA, Jeong YH, Marcucci R, Siller-Matula JM, Mehran R, Neumann FJ, Pereira NL, Rizas KD, Rollini F, So DYF, Stone GW, Storey RF, Tantry US, Berg JT, Trenk D, Valgimigli M, Waksman R, Sibbing D. International consensus statement on platelet function and genetic testing in percutaneous coronary intervention: 2024 update. JACC Cardiovasc Interv 2024;17:2639–2663. 39603778 10.1016/j.jcin.2024.08.027

[5] Galli M, Franchi F, Rollini F, Been L, Jaoude PA, Rivas A, Zhou X, Jia S, Maaliki N, Lee CH, Pineda AM, Suryadevara S, Soffer D, Zenni MM, Geisler T, Jennings LK, Bass TA, Angiolillo DJ. Platelet P2Y12 inhibiting therapy in adjunct to vascular dose of rivaroxaban or aspirin: a pharmacodynamic study of dual pathway inhibition vs. dual antiplatelet therapy. Eur Heart J Cardiovasc Pharmacother 2022;8:728–737. 35353154 10.1093/ehjcvp/pvac022

[6] Galli M, Rollini F, Been L, Zenni MM, Angiolillo DJ, Franchi F. Impact of diabetes mellitus on the pharmacodynamic effects of prasugrel and ticagrelor after switching from clopidogrel in patients with coronary artery disease. J Thromb Thrombolysis 2022;54:461–469. 36048358 10.1007/s11239-022-02696-4

[7] Chan MV, Chen M-H, Thibord F, Nkambule BB, Lachapelle AR, Grech J, Schneider ZE, Wallace de Melendez C, Huffman JE, Hayman MA, Allan HE, Armstrong PC, Warner TD, Johnson AD. Factors that modulate platelet reactivity as measured by 5 assay platforms in 3429 individuals. Res Pract Thromb Haemost 2024;8:102406. 38813256 10.1016/j.rpth.2024.102406 PMC11135030

[8] Breet NJ, Sluman MA, van Berkel MA, van Werkum JW, Bouman HJ, Harmsze AM, Kelder JC, Zijlstra F, Hackeng CM, ten Berg JM. Effect of gender difference on platelet reactivity. Neth Heart J 2011;19:451–457. 21901505 10.1007/s12471-011-0189-y PMC3203982

[9] Galli M, Terracina S, Schiera E, Mancone M, Frati L, Angiolillo DJ, Pulcinelli FM. Interindividual variability in platelet reactivity among individuals with or without antiplatelet therapy: results from a large tertiary care hospital. J Thromb Thrombolysis 2025;58:71–83. 39242457 10.1007/s11239-024-03022-w PMC11762593

[10] Kelton JG, Powers P, Julian J, Boland V, Carter CJ, Gent M, Hirsh J. Sex-related differences in platelet aggregation: influence of the hematocrit. Blood 1980;56:38–41. 7388180

[11] Cattaneo M, Lecchi A, Zighetti ML, Lussana F. Platelet aggregation studies: autologous platelet-poor plasma inhibits platelet aggregation when added to platelet-rich plasma to normalize platelet count. Haematologica 2007;92:694–697. 17488697 10.3324/haematol.10999

[12] Kubota K, Shirakura T, Orui T, Muratani M, Maki T, Tamura J, Morita T. [Changes in the blood cell counts with aging]. Nihon Ronen Igakkai Zasshi 1991;28:509–514. 1942631 10.3143/geriatrics.28.509

[13] Aradi D, Kirtane A, Bonello L, Gurbel PA, Tantry US, Huber K, Freynhofer MK, ten Berg J, Janssen P, Angiolillo DJ, Siller-Matula JM, Marcucci R, Patti G, Mangiacapra F, Valgimigli M, Morel O, Palmerini T, Price MJ, Cuisset T, Kastrati A, Stone GW, Sibbing D. Bleeding and stent thrombosis on P2Y12-inhibitors: collaborative analysis on the role of platelet reactivity for risk stratification after percutaneous coronary intervention. Eur Heart J 2015;36:1762–1771. 25896078 10.1093/eurheartj/ehv104

[14] Galli M, Benenati S, Capodanno D, Franchi F, Rollini F, D'Amario D, Porto I, Angiolillo DJ. Guided versus standard antiplatelet therapy in patients undergoing percutaneous coronary intervention: a systematic review and meta-analysis. Lancet 2021;397:1470–1483. 33865495 10.1016/S0140-6736(21)00533-X

[15] Vrints C, Andreotti F, Koskinas KC, Rossello X, Adamo M, Ainslie J, Banning AP, Budaj A, Buechel RR, Chiariello GA, Chieffo A, Christodorescu RM, Deaton C, Doenst T, Jones HW, Kunadian V, Mehilli J, Milojevic M, Piek JJ, Pugliese F, Rubboli A, Semb AG, Senior R, ten Berg JM, Van Belle E, Van Craenenbroeck EM, Vidal-Perez R, Winther S, Borger M, Gudmundsdóttir IJ, Knuuti J, Ahrens I, Böhm M, Buccheri S, Capodanno D, Christiansen EH, Collet JP, Dickstein K, Eek C, Falk V, Henriksen PA, Ibanez B, James S, Kedev S, Køber L, Kyriakou M, Magavern EF, McInerney A, McEvoy JW, Mersha CO, Mihaylova B, Mindham R, Neubeck L, Neumann FJ, Nielsen JC, Paolisso P, Paradies V, Pasquet AA, Piepoli M, Prescott E, Rakisheva A, Rocca B, Ruel M, Sandner S, Saraste A, Szummer K, Vaartjes I, Wijns W, Windecker S, Witkowsky A, Zdrakovic M, Zeppenfeld K, Shuka N, Bouraghda MA, Hayrapetyan HG, Reinstadler SJ, Musayev O, De Pauw M, Kušljugić Z, Gelev V, Skoric B, Karakyriou M, Kovarnik T, Nielsen LH, Abdel-Aziz IS, Ainla T, Porela P, Benamer H, Nadaraia K, Richardt G, Papafaklis MI, Becker D, Gudmundsdóttir IJ, Wolak A, Riccio C, Zholdin BK, Elezi S, Abilova S, Mintale I, Allam B, Badarienė J, Pereira B, Dingli P, Revenco V, Bulatovic N, Benouna EGM, Dedic A, Mitevska I, Angel K, Bryniarski K, Luz AMC, Popescu BA, Bertelli L, Beleslin BD, Hudec M, Fras Z, Freixa-Pamias R, Holm A, Jeger R, Marjeh MYB, Hammami R, Aytekin V, Nesukay EG, Swanson N, Shek AB. 2024 ESC guidelines for the management of chronic coronary syndromes. Eur Heart J 2024;45:3415–3537. 39210710 10.1093/eurheartj/ehae177

[16] Occhipinti G, Laudani C, Galli M, Ortega-Paz L, Kunadian V, Mendieta G, Rinaldi R, Andreotti F, Mehran R, López-Sobrino T, Capodanno D, Angiolillo DJ, Sabatè Tenas M, Brugaetta S. Sex differences in dual antiplatelet therapy de-escalation strategies after percutaneous coronary intervention: a network meta-analysis. Eur Heart J; 10.1093/eurheartj/ehaf473. Published online ahead of print 11 July 2025. 40643264

[17] Valgimigli M, Gragnano F, Branca M, Franzone A, Baber U, Jang Y, Kimura T, Hahn JY, Zhao Q, Windecker S, Gibson CM, Kim BK, Watanabe H, Song YB, Zhu Y, Vranckx P, Mehta S, Hong SJ, Ando K, Gwon HC, Serruys PW, Dangas GD, McFadden EP, Angiolillo DJ, Heg D, Jüni P, Mehran R. P2y12 inhibitor monotherapy or dual antiplatelet therapy after coronary revascularisation: individual patient level meta-analysis of randomised controlled trials. BMJ 2021;373:n1332. 34135011 10.1136/bmj.n1332 PMC8207247

